# The struggle to differentiate inflammation from infection in severely burned patients: time to send better biomarkers into the arena?

**DOI:** 10.1186/s13054-016-1194-8

**Published:** 2016-01-20

**Authors:** Patrick M. Honore, Herbert D. Spapen

**Affiliations:** ICU Department, Universitair Ziekenhuis Brussel, Vrije Universiteit Brussel, Brussels, Belgium

We read with great interest the highly educational review by Rowan et al. on wound healing and treatment of burn patients [1]. As discussed in detail by the authors, burn injury is inextricably linked with some states of inflammation. Acute-phase activation of crucial immune processes is imperative for survival and initiation of wound healing, otherwise inflammation may become detrimental when caused by intercurrent infection or diseases such as sepsis or pancreatitis. The bedside clinician is often faced with the problem of differentiating between burn- or infection-related inflammation. For this purpose, poorly specific markers such as C-reactive protein (CRP) and white blood cell (WBC) count are still frequently used. In recent years, however, procalcitonin (PCT) has emerged as a more optimal biomarker for diagnosis and prognosis of infection in severely burned patients [2]. Monitoring PCT levels over time could also steer antibiotic treatment, minimize antibiotic overuse, and reduce the risk of developing multi-drug resistant bacterial infections. Yet, due to limited access and cost, PCT has not supplanted CRP in many burn centers. Moreover, recent prospective studies have challenged the utility of the PCT assay and proposed other potentially valid biomarkers for detecting sepsis in burn injury. Paratz et al. [3] found that plasma levels of the N-terminal fragment of the pro-hormone brain-type natriuretic peptide better predicted early sepsis than PCT. Cakır Madenci et al. [4] reported that soluble CD14 subtype (presepsin) had similar diagnostic and prognostic accuracy as CRP and PCT. However, sepsis patients had significantly different presepsin, CRP, and WBC count but not PCT levels on the first day of sepsis compared with previous days. This preliminary experience needs confirmation in a larger patient cohort. In addition, the clinical usefulness of any novel biomarker must be assessed in specific subgroups (e.g., patients undergoing extracorporeal treatment) [5].

## Abbreviations

CRP: C-reactive protein
PCT: procalcitonin
WBC: white blood cell
